# miR-338-3p functions as a tumor suppressor in gastric cancer by targeting PTP1B

**DOI:** 10.1038/s41419-018-0611-0

**Published:** 2018-05-09

**Authors:** Feng Sun, Mengchao Yu, Jing Yu, Zhijian Liu, Xinyan Zhou, Yanqing Liu, Xiaolong Ge, Haidong Gao, Mei Li, Xiaohong Jiang, Song Liu, Xi Chen, Wenxian Guan

**Affiliations:** 1 Department of General Surgery, Drum Tower Hospital, Medical School of Nanjing University, 321 Zhongshan Road, Nanjing, Jiangsu 210008 China; 2 State Key Laboratory of Pharmaceutical Biotechnology, Jiangsu Engineering Research Center for MicroRNA Biology and Biotechnology, NJU Advanced Institute for Life Sciences (NAILS), School of Life Sciences, Nanjing University, 163 Xianlin Road, Nanjing, Jiangsu 210046 China; 3 Department of General Surgery, Sir Run Run Shaw Hospital, School of Medicine, Zhejiang University, East Qingchun Road, Hangzhou, 310016 China; 4 Medical School of Nanjing University, 22 Hankou Road, Nanjing, Jiangsu 210093 China

## Abstract

Gastric cancer (GC) is one of the most common malignant tumors and peritoneal metastasis is the primary cause for advanced GC’s mortality. Protein-tyrosine phosphatase 1B (PTP1B) functions as an oncogene and involves in carcinogenesis and cancer dissemination. However, the function and regulation of PTP1B in GC remain poorly understood. In this study, we found that PTP1B was upregulated in GC tissues and overexpression of PTP1B in vitro promoted cell migration and prevented apoptosis. Then, we predicted that PTP1B was a target of miR-338-3p and we revealed an inverse correlation between miR-338-3p levels and PTP1B protein levels in GC tissues. Next, we verified that PTP1B was inhibited by miR-338-3p via direct targeting to its 3′-untranslated regions. Moreover, overexpression of miR-338-3p in vitro attenuated GC cell migration and promoted apoptosis, and these effects could be partially reversed by reintroduction of PTP1B. Finally, we established an orthotopic xenograft model and a peritoneal dissemination model of GC to demonstrate that miR-338-3p restrained tumor growth and dissemination in vivo by targeting PTP1B. Taken together, our results highlight that PTP1B is an oncogene and is negatively regulated by miR-338-3p in GC, which may provide new insights into novel molecular therapeutic targets for GC.

## Introduction

There exist almost one million new cases of gastric cancer (GC) every year worldwide and half of these occur in Eastern Asia, China in particular. Although the incidence of GC has declined over the years, it remains the fifth most common cancer and the third leading cause of cancer-related death in the world^1^. The poor prognosis of GC is mainly attributed to tumor metastasis and recurrence. Peritoneal metastasis is the most frequent dissemination pattern and accounts for over 40 percent among all recurrence cases^2^. Once peritoneal recurrence occurs, the mean survival time is only 18 months^3^. Therefore, a better understanding of molecular mechanisms underlying tumor development, especially metastasis, is necessary for more effective therapeutic strategies.

As we all know, cancer metastasis is a multi-step process facilitated by several cancer-related molecular factors^4^. Take peritoneal dissemination of GC as an example. Formation of metastasis involves detachment from the primary tumor, migration, and attachment to mesothelial cells, invasion into subperitoneal tissue, and proliferation with angiogenesis^5^. Although a large number of studies have been carried out to elucidate the molecular mechanisms involved in each step, the complicated underlying mechanisms remain unclear. Protein-tyrosine phosphatase 1B (PTP1B), encoded by the *PTPN1* gene in human, is a member of protein-tyrosine phosphatase (PTP) family^6^. PTP1B was well known initially, owing to direct regulation of the insulin and the leptin signaling pathway; thus, it was considered to be a promising therapeutic target for type II diabetes and obesity^7^. Recently, more attention has been attracted to the dual role of PTP1B in human cancer^8–10^. PTP1B was initially regarded as a tumor suppressor gene, because it might terminate signal responses of oncogenic kinases through the dephosphorylation. The genetic deletion of PTP1B in p53-null mice increased the susceptibility of lymphomagenesis^11^. In esophageal cancer, the expression of PTP1B was inhibited in tumor tissues compared with that in adjacent normal tissues^12^. In contrast, more recent studies have shown that PTP1B was commonly overexpressed in tumor tissues and it had a positive role in tumor development and progression, such as breast, prostate, colorectal, lung, and hepatocellular cancer^13–15^. PTP1B was well studied in breast cancer in the aspect of synergizing with the ErbB2 oncogene^16–18^. With respect to GC, PTP1B expression was greatly elevated in GC tissues according to a recent research^19^. The elevated PTP1B expression promoted GC cell proliferation both in vitro and in vivo via activation of protein kinase B (AKT), extracellular signal-regulated kinase (ERK) 1/2, and focal adhesion kinase signaling pathway^19^. Its oncogenic role in GC was further confirmed by another study and PTP1B overexpression was associated with poor survival of patients with GC^20^.

Given the increasing appreciation of PTP1B as a crucial oncogene, the mechanisms underlying the upregulation of PTP1B expression in cancer need to be fully investigated. MicroRNAs (miRNAs) are short non-coding RNAs molecules, with 19–23 nucleotide in length. miRNAs mainly function in negative regulation of gene expression by binding to their target sites, usually in the 3′-untranslated regions (UTRs) of mRNAs. Once binding to its target mRNA, a miRNA typically leads to translational repression or degradation of the mRNA^21^. An extensive literature shows that miRNAs are implicated in various physiological and pathological processes, with no exception of human cancers^22, 23^. In the initiation and progression of human cancers, miRNAs can act as either oncomiRs or tumor suppressors via interacting with oncogenes or tumor suppressor genes^24^. miR-338-3p was initially identified as a brain specifically expressed miRNA, which participated in the formation of basolateral polarity and regulation of axonal respiration^25, 26^. Recent research expanded its function into the field of the tumor. miR-338-3p was reported to be a tumor suppressor miRNA through inhibiting the proliferation, migration, and invasion in hepatocellular^27^, breast^28^, and ovarian cancer^29^. The expression of miR-338-3p was also downregulated in GC tissues and the degree of decline correlated with the tumor stage, which suggested that miR-338-3p might be involved in the progression of GC^30^. Its tumor suppression effect was confirmed by Huang et al.^31^ who demonstrated that miR-338-3p inhibited the epithelial–mesenchymal transition (EMT) of GC cells. Nevertheless, the substantial mechanism through which miR-338-3p participates in GC progression, especially peritoneal metastasis, remains to be answered.

In this study, we first assumed that the expression of PTP1B was posttranscriptionally regulated to some extent in GC based on disagreements between the expression of protein and mRNA in several GC cases. Then we discovered that miR-338-3p directly targeted PTP1B, which further suppressed migration, and promoted apoptosis in GC cells. Finally, the result of in vivo experiments further validated miR-338-3p inhibited tumor growth and peritoneal dissemination of GC via PTP1B.

## Results

### PTP1B was upregulated and it functioned as an oncogene in GC

**Fig. 1:**
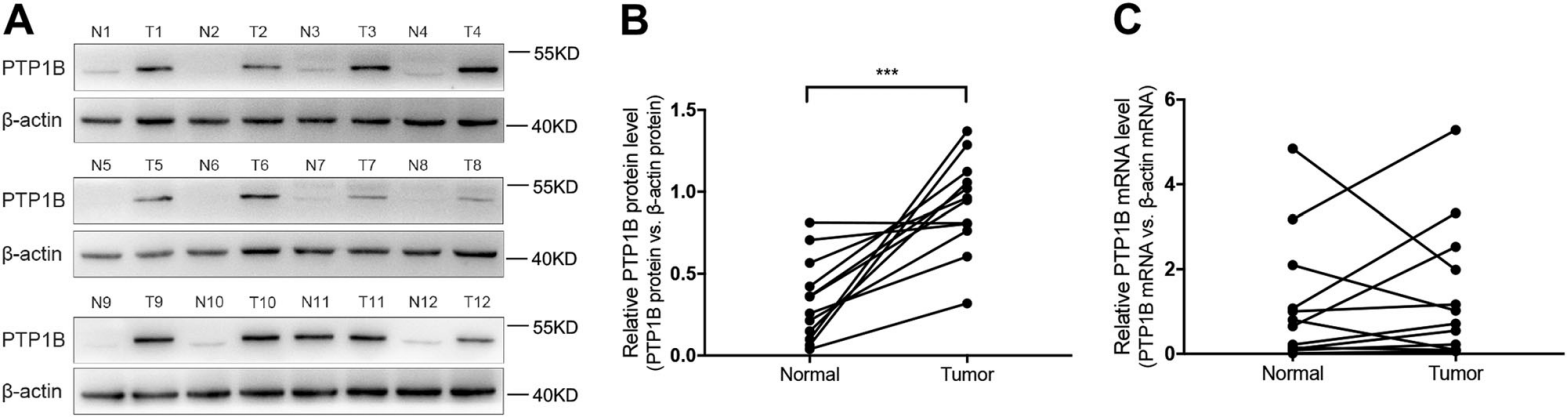
PTP1B is upregulated in human GC tissues. **a**,**b** Western blot analysis of PTP1B protein in 12 paired human GC (T) and adjacent normal tissue (N) specimens. **a** Representative images; **b** quantitative analysis. **c** qRT-PCR analysis of PTP1B mRNA levels in the same 12 pairs of GC and adjacent normal specimens. *n* = 12; *** *P* < 0.001; two-tailed Student’s *t*-test

**Fig. 2:**
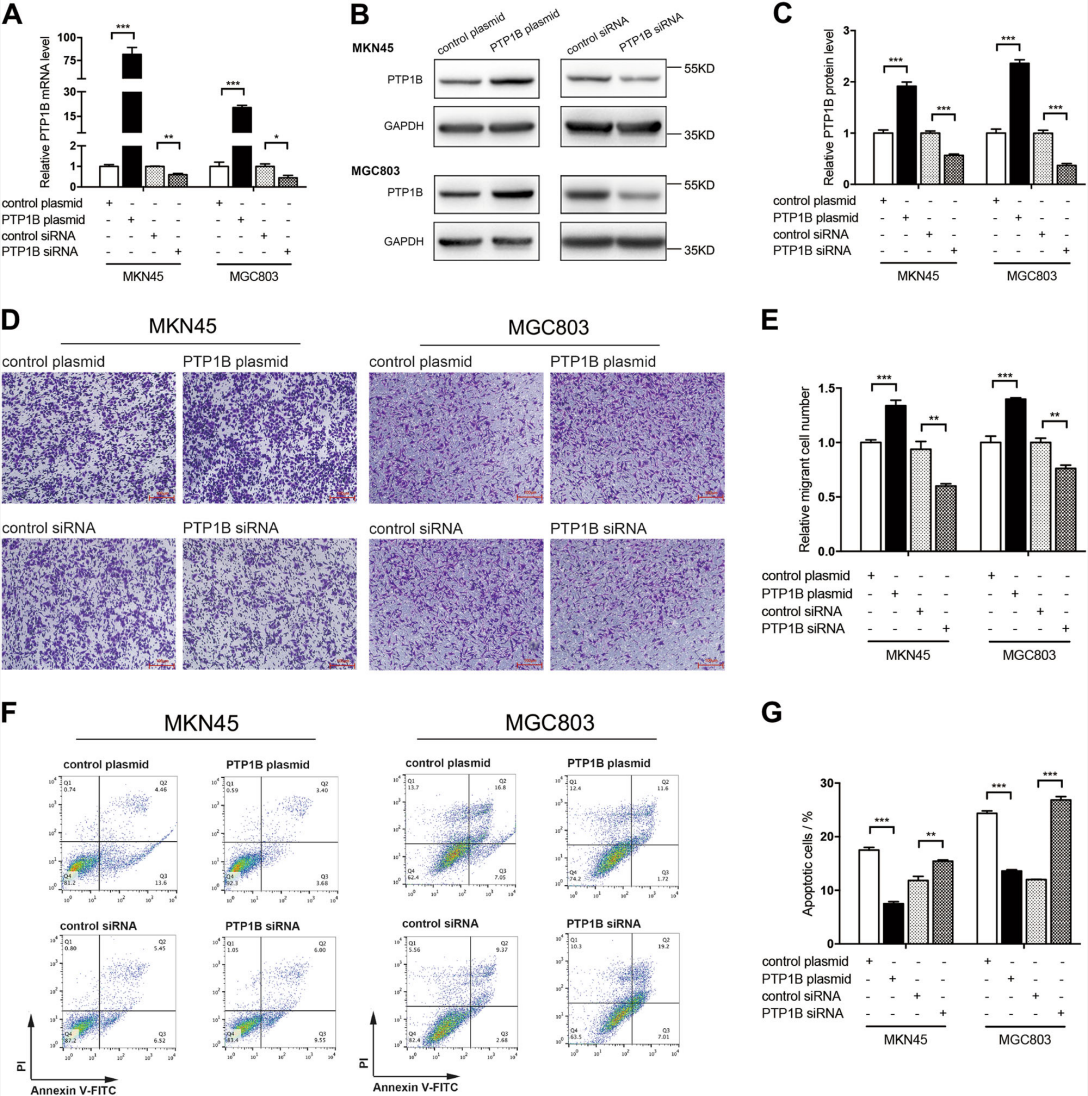
PTP1B functions as an oncogene to promote migration and inhibit apoptosis of GC cells. **a**–**c** The efficiency of PTP1B overexpression and knockdown in GC cells. **a** qRT-PCR analysis of PTP1B mRNA levels in MKN45 and MGC803 cells treated with PTP1B plasmid, control plasmid, PTP1B siRNA, or scrambled control siRNA. **b**,**c** Western blottings of PTP1B protein in MKN45 and MGC803 cells treated with PTP1B plasmid, control plasmid, PTP1B siRNA, or scrambled control siRNA. **b** Representative images; **c** quantitative analysis. **d**,**e** Transwell assays were performed after the transfection of MKN45 and MGC803 with PTP1B plasmid, control plasmid, PTP1B siRNA, and control siRNA. **d** Representative images; Scale bar, 100 μm. **e** Quantitative analysis. **f**,**g** The apoptosis assays were performed 24 h after the transfection of MKN45 and MGC803 cells with PTP1B plasmid, control plasmid, PTP1B siRNA, and control siRNA. **f** Representative images; **g** quantitative analysis. *n* = 3 independent repeats. Data are represented as mean ± SEM; *P < 0.05; **P < 0.01; ***P < 0.001; two-tailed Student’s *t*-test

To evaluate the expression of PTP1B in GC, we examined the PTP1B protein levels in 12 pairs of GC patient specimens and their corresponding adjacent normal tissues. We found that PTP1B protein levels were remarkably higher in GC tissues compared with those of the noncancerous counterparts (Fig. 1a, b). PTP1B mRNA levels were also detected in the same specimens described above. Interestingly, in 12 pairs of tissue specimens, the mRNA levels of 5 GC tissues were nearly equal or even declined compared with those in adjacent normal tissues (Fig. 1c). In addition, we also looked at the levels of PTP1B in human GC cell lines and human GC specimens from TCGA dataset. Results further verified that PTP1B was upregulated in GC (Additional file 1: Figure S1). We then tested the biological function of PTP1B in two GC cell lines (MKN45 and MGC803). Two biological actions (migration and apoptosis) were evaluated to show its effects on progression of GC. The migration was determined by Transwell migration assays, whereas cell apoptosis was tested by Annexin V-fluorescein isothiocyanate (FITC) Apoptosis Detection Kit I. PTP1B-overexpression plasmid and small interfering RNA (siRNA) were used to enhance or silence PTP1B protein expression, respectively (Fig. 2a–c). Results showed that PTP1B overexpression promoted the migration and suppressed the apoptosis of GC cells, whereas PTP1B knockdown generated an opposite effect (Fig. 2d–g).

### Prediction of PTP1B as a target gene of miR-338-3p

As described above, we found an interesting phenomenon that PTP1B mRNA levels and protein levels were inconsistently expressed in human GC tissues. Hence, we predicted an important impact of posttranscriptional regulation on the expression of PTP1B protein levels in GC tissues.

**Fig. 3:**
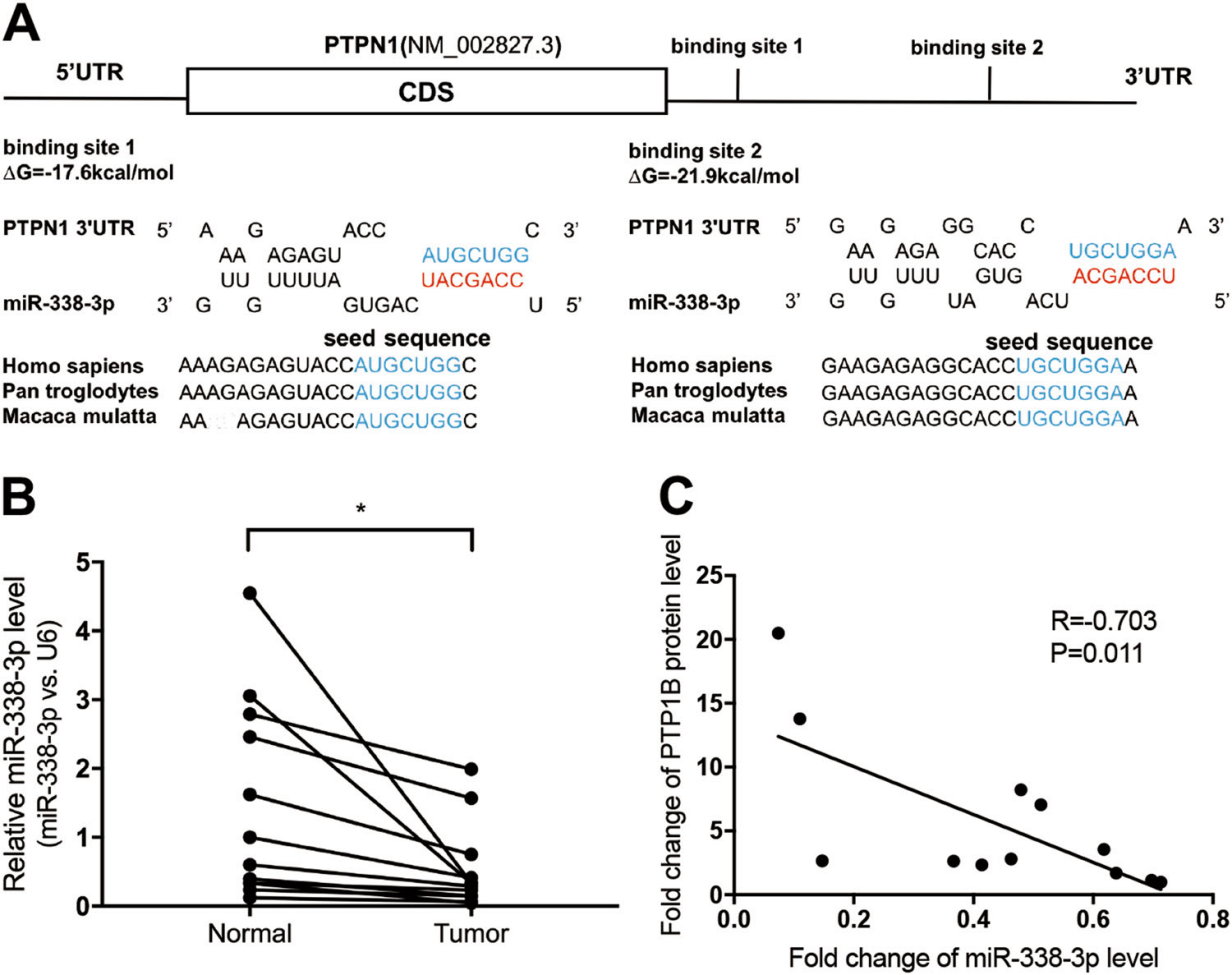
Prediction of PTP1B as a miR-338-3p target. **a** Schematic of the hypothetical duplex formed by the interaction between the binding sites in the PTPN1 3′-UTR and miR-338-3p. The calculated free energy values of the hybrid are shown. **b** qRT-PCR analysis of miR-338-3p levels in the same specimens (*n* = 12; two-tailed Student’s *t*-test). **c** Pearson’s correlation scatter plot of the fold changes of miR-338-3p and PTP1B protein in human GC tissues (*n* = 12; Pearson’s correlation test). *P < 0.05

It is well known that miRNAs are widely implicated in posttranscriptional regulation, so we supposed that PTP1B protein levels in GC might be regulated by miRNAs. We searched two databases with different algorithms, TargetScan and miRanda, to identify potential miRNAs which could target PTP1B. Among these candidates, miR-338-3p was predicted by both bioinformatics databases, so it was selected for the further analysis. As shown in Fig. 3a, two potential binding sites of miR-338-3p were identified at the 3′-UTR of PTP1B. The minimum free energy values of the hybrid were − 17.6 and − 21.9 kcal/mol, respectively, both of which were well within the range of genuine miRNA-target pairs (Fig. 3a).

To evaluate the potential clinical relevance of miR-338-3p and PTP1B expression, we first examined the expression of miR-338-3p in the same 12 pairs of tissue specimens and results showed that miR-338-3p levels were lower in GC tissues (Fig. 3b). Then, we used Pearson’s correlation scatter plots to illustrate the inverse correlation between miR-338-3p and PTP1B protein levels (Fig. 3c). The expression of miR-338-3p showed a significant negative correlation with PTP1B protein levels (*R* = − 0.703).

### PTP1B was a direct downstream target of miR-338-3p

**Fig. 4:**
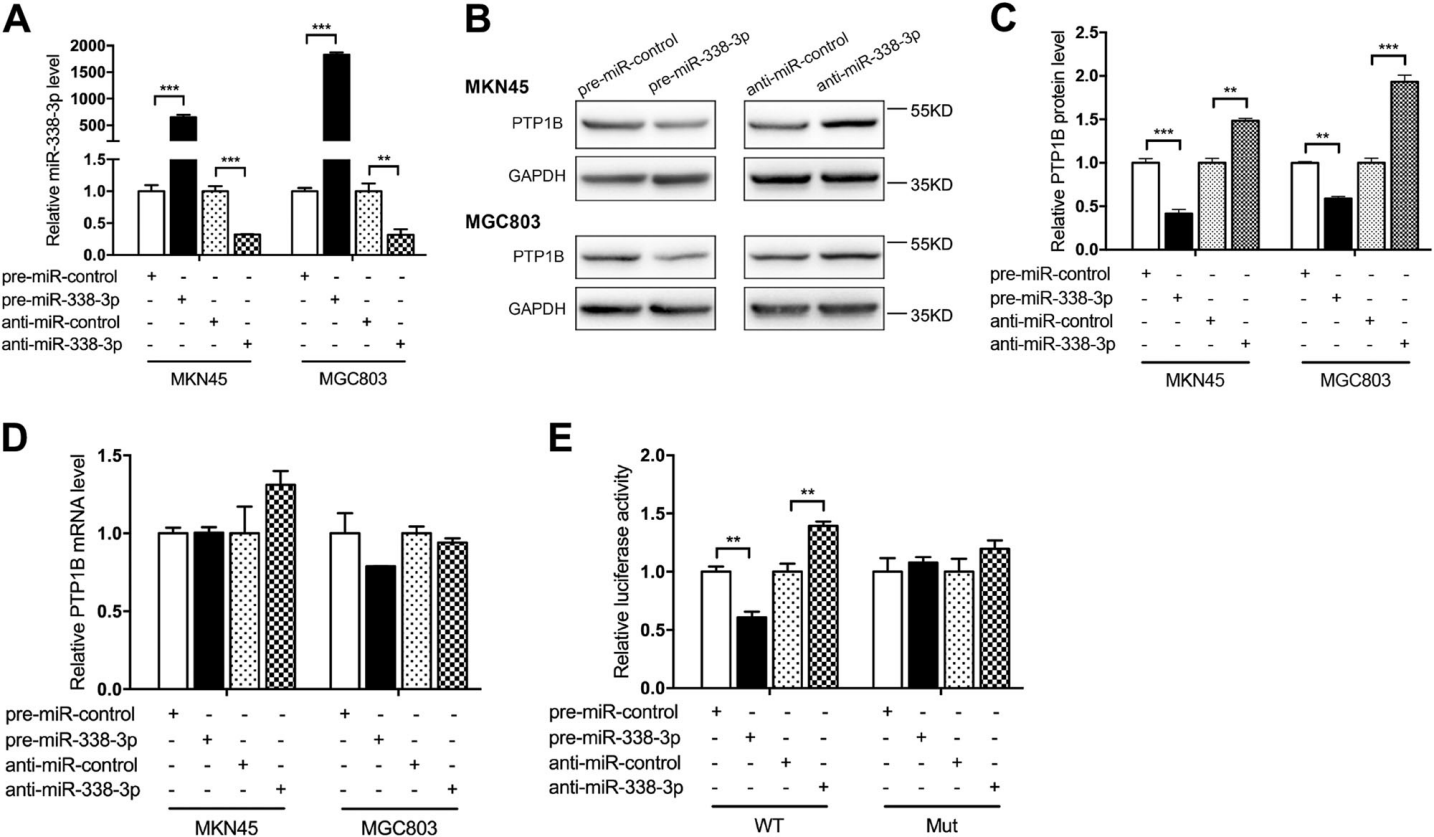
PTP1B is a direct target of miR-338-3p. **a–d** MKN45 and MGC803 cells were transfected with miR-338-3p mimic (pre-miR-338-3p), miR-338-3p inhibitor (anti-miR-338-3p), or scrambled negative control RNA. After 48 h, cells were collected for total RNA and protein extraction. **a** qRT-PCR analysis of miR-338-3p levels in MKN45 and MGC803 cells. **b**,**c** Western blot analysis of PTP1B protein in MKN45 and MGC803 cells. **b** Representative images; **c** quantitative analysis. **d** qRT-PCR analysis of PTP1B mRNA levels in MKN45 and MGC803 cells. **e** The relative luciferase activities in MKN45 co-transfected with luciferase reporter plasmids containing wild-type (WT) or mutant (Mut) PTP1B 3′-UTR, and miR-338-3p mimic, miR-338-3p inhibitor, or scrambled negative control RNA. *n* = 3 independent repeats. Data are represented as mean ± SEM; *P < 0.05; **P < 0.01; ***P < 0.001; two-tailed Student’s *t*-test

We first tested the ability of miR-338-3p to regulate PTP1B protein expression in human GC cell lines. miR-338-3p mimic and miR-338-3p inhibitor were used to overexpress or knock down miR-338-3p (Fig. 4a). When transfected with miR-338-3p mimic, cells showed its PTP1B protein level significantly decreased (Fig. 4b, c). Conversely, suppression of the endogenous miR-338-3p remarkably increased the PTP1B protein level (Fig. 4b, c). PTP1B mRNA levels were also detected, but we did not see any alteration after either overexpression or knockdown of miR-338-3p (Fig. 4d). To verify these findings, we repeated the above experiments in another GC cell lines (MGC803) and observed similar results (Fig. 4a–d). Moreover, we also used miR-338-3p overexpression lentivirus to simulate high expression of endogenous miR-338-3p (Additional file 2: Figure S2a). As a result, PTP1B protein levels were significantly suppressed (Additional file 2: Figure S2b and c), while PTP1B mRNA levels made no obvious change (Additional file 2: Figure S2d). In addition, we used another GC cell line (MGC803) to verify these findings (Additional file 2: Figure S2a-d).

Next, to examine whether miR-338-3p directly bound to the PTP1B 3′-UTR, we cloned the predicted miR-338-3p-binding sites into the luciferase reporter vector, constructing wild-type luciferase reporter vector (pMIR-PTP1B-wt-3′-UTR). Concomitantly, the putative miR-338-3p-binding sites were mutated to construct a mutant luciferase reporter vector (pMIR-PTP1B-mut-3′-UTR). Each vector was co-transfected with miR-338-3p mimic, miR-338-3p inhibitor, or corresponding negative control RNA into MKN45 cells, and luciferase signal was determined by luciferase assays. As expected, overexpression of miR-338-3p significantly reduced the luciferase activity of wild-type reporter vector, whereas this effect was abolished in the mutant reporter vector (Fig. 4e). Conversely, knockdown of miR-338-3p significantly elevated the luciferase activity of wild-type reporter vector, but not of mutant reporter vector (Fig. 4e). These data indicated that PTP1B was the direct target of miR-338-3p.

Taken together, these results strongly suggested that miR-338-3p downregulated PTP1B protein level through directly binding to its 3′-UTR.

### miR-338-3p attenuated GC cell migration and promoted apoptosis by targeting PTP1B

**Fig. 5:**
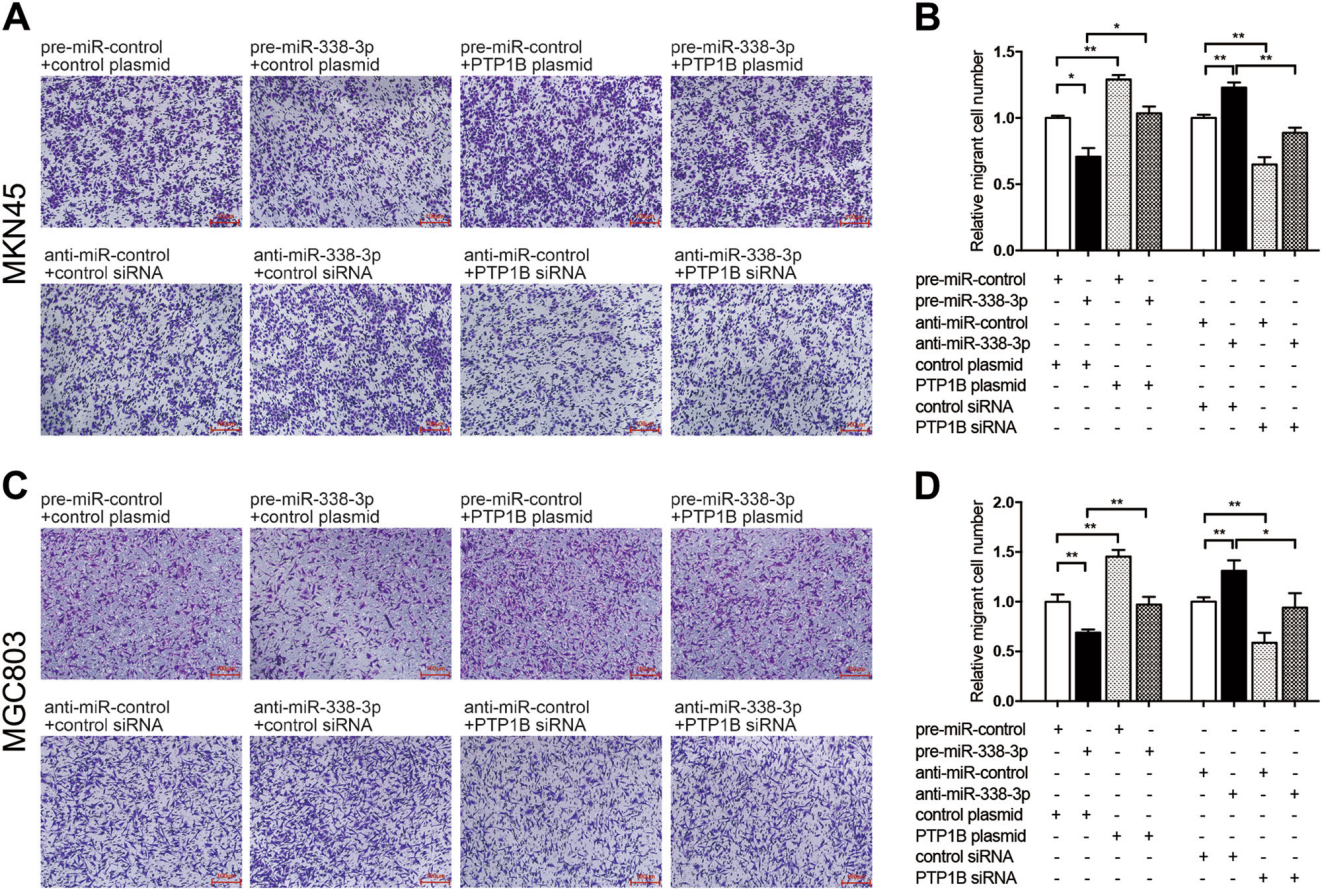
miR-338-3p suppresses GC cell migration by targeting PTP1B. **a**–**d** The migration assays (Transwell) were performed in MKN45 and MGC803 cells that were transfected with control mimic plus control plasmid, miR-338-3p mimic plus control plasmid, control mimic plus PTP1B-overexpression plasmid, miR-338-3p mimic plus PTP1B-overexpression plasmid, or with control inhibitor plus control siRNA, miR-338-3p inhibitor plus control siRNA, control inhibitor plus PTP1B siRNA, miR-338-3p inhibitor plus PTP1B siRNA. **a**,**c** Representative images; Scale bar, 100 μm. **b**,**d** Quantitative analysis. *n* = 3 independent repeats. Data are represented as mean ± SEM; *P < 0.05; **P < 0.01; two-tailed Student’s *t*-test

Migration was a crucial process in tumor metastasis and PTP1B was demonstrated to advance this process. Thus, we investigated the biological significance of miR-338-3p in MKN45 cells migration. Less migration was observed in MKN45 cells transfected with miR-338-3p mimic (Fig. 5a, b); conversely, cells transfected with miR-338-3p inhibitor showed increased migration (Fig. 5a, b). The above-mentioned results showed that miR-338-3p and PTP1B had opposing effects on cell migration. Although PTP1B was one of the direct targets of miR-338-3p, whether miR-338-3p affected this biological process by inhibiting PTP1B remains to be investigated. Thus, we co-transfected MKN45 cells with miR-338-3p mimic and PTP1B-overexpression plasmid cell migration capacity was rescued compared with those transfected with miR-338-3p mimic alone (Fig. 5a, b). These findings illustrated that restoration of PTP1B expression can reverse the negative effect of miR-338-3p on cell migration. Conversely, cells co-transfected with miR-338-3p inhibitor and PTP1B siRNA could impair cell migration compared with those transfected with miR-338-3p inhibitor alone (Fig. 5a, b), suggesting that knockdown of PTP1B expression could reverse the positive effect of anti-miR-338-3p on cell migration. The above experiments were repeated in MGC803 and observed similar results (Fig. 5c, d). To validate the above results, we performed another migration assay (scratch-wound assays) and got a consistent result (Additional file 3: Figure S3a-h). Taken together, miR-338-3p exerted its tumor suppressor function by targeting PTP1B in GC cells.

**Fig. 6:**
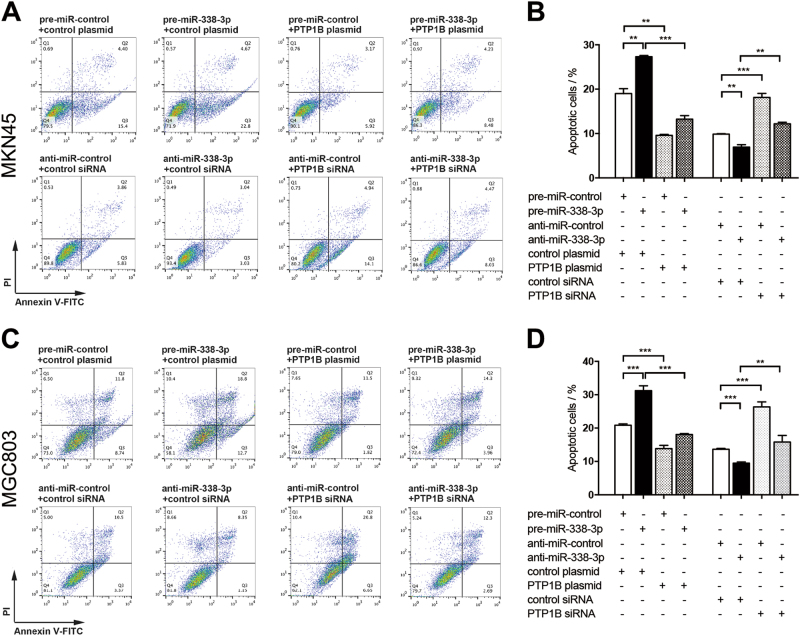
miR-338-3p promotes GC cell apoptosis by targeting PTP1B. **a**–**d** Cell apoptosis assays were performed 24 h after the transfection of MKN45 and MGC803 cells with control mimic plus control plasmid, miR-338-3p mimic plus control plasmid, control mimic plus PTP1B-overexpression plasmid, miR-338-3p mimic plus PTP1B-overexpression plasmid, or with control inhibitor plus control siRNA, miR-338-3p inhibitor plus control siRNA, control inhibitor plus PTP1B siRNA, miR-338-3p inhibitor plus PTP1B siRNA. **a**,**c** Representative images; **b**,**d** quantitative analysis. *n* = 3 independent repeats. Data are represented as mean ± SEM; **P < 0.01; ***P < 0.001; two-tailed Student’s *t*-test

Anti-apoptotic capacity is another critical ability to form peritoneal metastasis of GC. Cell apoptosis assay was performed to measure apoptotic cells. Similar effects on apoptosis of the overexpression and knockdown of miR-338-3p were observed in MKN45 cells (Fig. 6a, b). Cell apoptosis triggered by miR-338-3p was partially repaired by transfection of PTP1B-overexpression plasmid (Fig. 6a, b). Likewise, cell apoptosis suppressed by anti-338-3p were reversed by transfection of PTP1B siRNA (Fig. 6a, b). We repeated the above experiments in MGC803 and observed similar results (Fig. 6c, d). To confirm the above results, we also examined the expression of cleaved Caspase-3 in cells with different treatment and got a consistent result (Additional file 4: Figure S4a-c). All of these results suggest that miR-338-3p involved in regulation of GC cell migration and apoptosis through silencing PTP1B.

The molecular mechanisms by which PTP1B overexpression led to increased migration and reduced apoptosis in GC were further investigated. mitogen-activate dprotein kinase (MAPK)/ERK and phosphotidyl inositol 3-kinase (PI3K)/AKT signaling pathways are two important transduction systems in the cell, which have critical roles in cell proliferation, migration, and apoptosis^32, 33^. Thus, we investigated whether PTP1B produced its effect by activating these pathways. Evaluation showed that PTP1B overexpression elevated the level of phosphorylated AKT (p-AKT) and phosphorylated ERK1/2 (p-ERK1/2) in MKN45 and MGC803 cells (Additional file 5: Figure S5a-c). These results suggest that Akt and ERK signaling pathways may be implicated in the process of PTP1B-promoting GC cells migration and inhibiting apoptosis.

### miR-338-3p inhibited tumor growth in vivo by targeting PTP1B

Finally, we demonstrated the effects of miR-338-3p and PTP1B on the growth of GC in vivo by establishing the GC orthotopic transplantation model in Balb/c nude mice using MKN45 cells. In order to monitor tumor growth in a non-invasive way, we constructed MKN45-Luc cells lines, which stably express luciferase using firefly luciferase lentiviruses. As shown in former studies, there exists a good correlation between bioluminescent intensity and the number of bioluminescent cells in vivo^34–36^, so we can determine tumor size by quantifying the bioluminescent signals emitted from tumor-bearing nude mice.

**Fig. 7:**
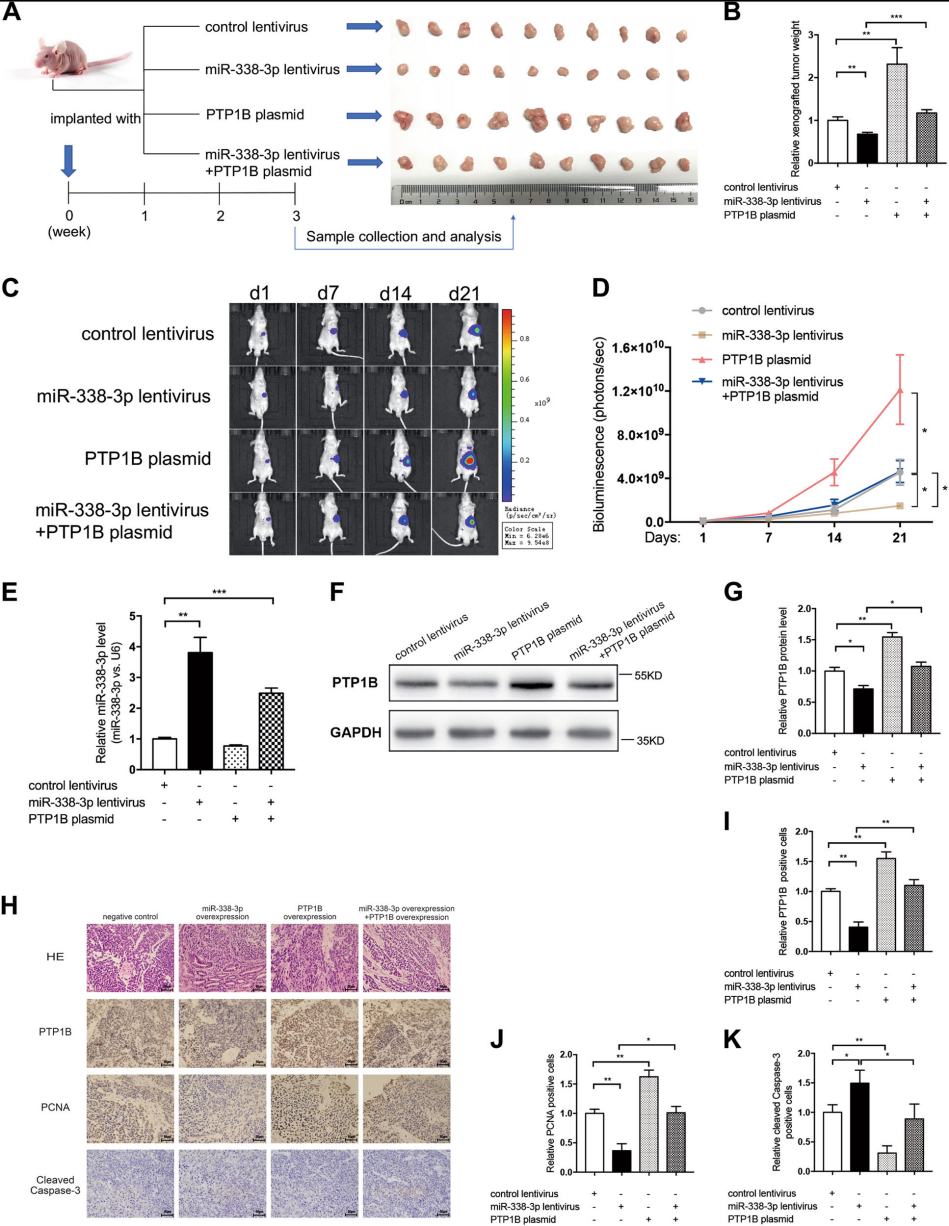
miR-338-3p suppresses GC growth in vivo by targeting PTP1B. **a** Flowchart of the experimental plan and representative images of xenograft tumors. MKN45 cells were infected with lentiviruses expressing firefly luciferase to construct MKN45-Luc cell line. MKN45-Luc cells were further infected with control lentivirus, miR-338-3p lentivirus, PTP1B plasmid, or miR-338-3p lentivirus plus PTP1B plasmid. Then, cells (1 × 10^6^ cells) were implanted into the subserosal area of the stomach of nude mice (10 mice per group) and the tumor growth was evaluated on day 21 after cell implantation. **b** Quantitative analysis of the tumor weight. **c**,**d** Bioluminescence imaging of tumors on day 1, 7, 14, and 21 after implantation of GC cells. **c** Representative images; **d** quantitative analysis. **e** qRT-PCR analysis of miR-338-3p levels in xenograft tumors. **f**,**g** Western blotting analysis of PTP1B protein levels in xenograft tumors. **f** Representative image; **g** quantitative analysis. **h**–**k** H&E and immunohistochemical staining for PTP1B, PCNA, and cleaved Caspase-3 in xenograft tumors. **h** Representative images; scale bar, 50 μm. **i**–**k** Quantitative analysis. *n* = 10 mice per group; data are represented as mean ± SEM; *p < 0.05; **p < 0.01; ***p < 0.001; two-tailed Student’s *t*-test

The MKN45-Luc cells were further infected with miR-338-3p overexpression lentivirus, transfected with PTP1B-overexpression plasmid, or co-transfected with miR-338-3p overexpression lentivirus and PTP1B-overexpression plasmid, respectively. Next, we constructed the orthotopic xenograft model by injecting these treated MKN45-Luc cells into the subserosal area of the stomach. Bioluminescent imaging of xenograft tumors was performed weekly to monitor the cancer growth. Flowchart of the experimental plan and representative images of tumors from implanted mice were shown in Fig. 7a. At the end of the observation period, tumors were resected from mice and tumor weight was measured. We found that tumor growth was significantly inhibited in the miR-338-3p-overexpressing group, but increased in the PTP1B-overexpressing group (Fig. 7a, b). In addition, the mice weight was higher in groups with both miR-338-3p and PTP1B overexpression compared with that with miR-338-3p overexpression alone (Fig. 7a, b). From the bioluminescence images, we observed a substantial reduction of signal intensity in miR-338-3p-overexpressing group and a significant increase in PTP1B-overexpressing group (Fig. 7c, d). Xenografts with both miR-338-3p and PTP1B overexpression exhibited increased signal intensity compared with that with miR-338-3p overexpression (Fig. 7c, d), suggesting that PTP1B overexpression could attenuate the growth suppression induced by miR-338-3p. This is in keeping with the in vitro experiments. We next extracted and analyzed total RNA and protein from the tumors. Significant upregulation of miR-338-3p and downregulation of PTP1B protein levels were observed in the miR-338-3p-overexpressing group (Fig. 7e–g). Reintroduction of PTP1B could partially reverse the negative effect of miR-338-3p on PTP1B protein expression levels (Fig. 7f, g). The tumor tissues were further confirmed by hematoxylin and eosin (H&E and immunochemistry staining (Fig. 7h). Tumors from the miR-338-3p-overexpressing group had less PTP1B- and PCNA-positive cells, and more cleaved Caspase-3-positive cells, whereas tumors from the PTP1B-overexpressing group had more PTP1B- and PCNA-positive cells, and less cleaved Caspase-3-positive cells (Fig. 7h–k). Likewise, the reintroduction of PTP1B restored the effects of miR-338-3p on PTP1B, PCNA, and cleaved Caspase-3 expression levels (Fig. 7h–k).

To determine the possible downstream mechanisms of PTP1B, we performed the immunochemistry staining of p-AKT and p-ERK1/2. Both p-AKT and p-ERK1/2 levels were lower in tumors of the miR-338-3p-overexpressing group, whereas higher in tumors from the PTP1B-overexpressing group (Additional file 6: Figure S6a-c). Similarly, the reintroduction of PTP1B restored the inhibition of p-AKT and p-ERK1/2 expression levels affected by miR-338-3p (Additional file 6: Figure S6a–c).

### miR-338-3p inhibited tumor metastasis in vivo by targeting PTP1B

**Fig. 8:**
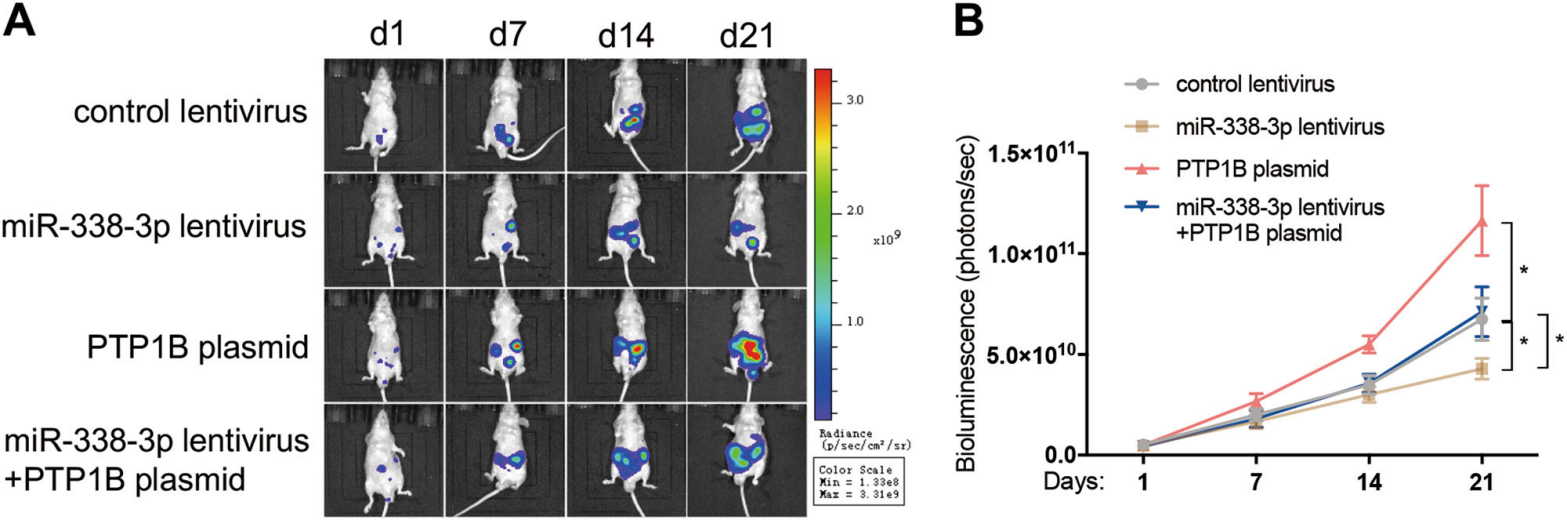
miR-338-3p suppresses peritoneal dissemination of GC in vivo by targeting PTP1B. **a**,**b** MKN45-Luc cells (5 × 10^6^ cells) infected with control lentivirus, miR-338-3p lentivirus, PTP1B plasmid, or miR-338-3p lentivirus plus PTP1B plasmid were injected into the left lower peritoneal cavity (i.p.) of nude mice (10 mice per group). Bioluminescence images of tumors were detected on day 1, 7, 14, and 21 after implantation of GC cells. **a** Representative images; **b** quantitative analysis. *n* = 10 mice per group; data are represented as mean ± SEM; *p < 0.05; two-tailed Student’s *t*-test

To further elucidate the effect of miR-338-3p on peritoneal metastasis of GC, we established a peritoneal dissemination model of GC by injecting MKN45-Luc cells into the peritoneal cavity. Mice were divided into four groups: control group, miR-338-3p-overexpressing group, PTP1B-overexpressing group, and both miR-338-3p and PTP1B-overexpression group. Bioluminescence images were also taken weekly from day 1 to day 21 post injection. Luminescence signals were remarkably lower in the miR-338-3p-overexpressing group, but strengthened in the PTP1B-overexpressing group (Fig. 8a, b). PTP1B overexpression in the miR-338-3p lentivirus-infected group reversed the suppression of formation of metastatic foci caused by miR-338-3p overexpression (Fig. 8a, b).

Together, these results suggested that miR-338-3p significantly inhibited tumor growth and peritoneal dissemination of GC in vivo by downregulating PTP1B expression.

## Discussion

As GC is usually asymptomatic in its early stages, many people are diagnosed with advanced cancer at the beginning. Peritoneal metastasis, which could lead to bowel obstruction and formation of malignant ascites, is the most common cause of mortality among patients with advanced GC^37, 38^. For these patients of whom the tumor is inoperable, chemotherapy serves as an appropriate treatment and hyperthermic intraperitoneal chemotherapy herald a promising future for the treatment of peritoneal metastasis^39, 40^. Recently, molecular targeted therapy has attracted increasing attention for its anti-cancer specificity, but it relies on the generation of more useful molecular targets. PTP1B was purified and characterized from human placenta in 1988^41, 42^. Since then, our views of this enzyme have evolved from a simple metabolic off-switch to a double-edged sword in oncogenesis^8, 9^. To date, it seems indisputable that PTP1B functions as an oncogene in the majority of solid tumor, and has a crucial role in tumor metastasis. Knockdown of PTP1B in colorectal cancer cell lines reduced cell adhesion, migration, and anoikis resistance^43^. The elevated PTP1B expression promoted the proliferation and metastasis of non-small cell lung cancer (NSCLC) cell lines through activating src and ERK1/2^44^. Consistently, deletion of PTP1B activity in vivo delayed the onset of breast cancer formation and reduced lung metastasis^45^. In this study, we showed that PTP1B was upregulated in GC tissues. Overexpression of PTP1B in vitro remarkably promoted cell migration and prevented apoptosis. Also, in vivo studies further confirmed the positive role of PTP1B in GC tumorigenesis and peritoneal dissemination. All of these results confirmed that PTP1B functioned as an oncogene in GC. What’s more, we observed an interesting result that in some GC cases, PTP1B mRNA levels and protein levels were inconsistent. This phenomenon led us to suppose that there could be a posttranscriptional mechanism involved in the regulation of PTP1B protein expression in GC.

As key posttranscriptional regulators, miRNAs are widely implicated in tumorigenesis and several stages of metastasis^46^. Bioinformatic analysis revealed that PTP1B may be the target gene of miR-338-3p. miR-338-3p is located at chromosome 17q25 within the eighth intron of the apoptosis-associated tyrosine kinase (*AATK*) gene^47^. Previous studies have demonstrated that reduced miR-338-3p was correlated with more advanced pathological stage, and lymph node metastasis in NSCLC and GC^31, 48^, indicating that miR-338-3p was involved in the progression of both primary tumors and metastatic disease. Recent studies showed that miR-338-3p acted as a tumor suppressor in GC by targeting zinc finger E-boxbinding protein 2, ADAM metallopeptidase domain 17, and P-Rex2a^30, 31, 49^. In our present study, we first demonstrated that miR-338-3p levels in GC specimens were negatively correlated with PTP1B protein levels. Then, we further discovered that miR-338-3p inhibited PTP1B expression by directly targeting the 3′-UTR of the PTP1B mRNA, and the regulatory relationships were validated in two GC cell lines. Subsequently, we revealed the crucial effects of miR-338-3p-driven suppression of PTP1B on GC cell migration and apoptosis, and tumor growth and peritoneal dissemination in xenograft mouse models. These results together demonstrated that miR-338-3p had a tumor-suppressive role in GC by inhibiting PTP1B.

As for the downstream mechanism by which PTP1B affected cell migration, apoptosis, and tumor growth, we examined the activation of PI3K/AKT and MAPK/ERK signaling pathways. According to the previous studies, both of these pathways had an important role in cell proliferation, and dissemination and survival^32^. PI3K/AKT pathway mainly promoted cell anabolism, whereas MAPK/ERK pathway was more active in cell proliferation and invasion^50^. In our study, we showed that PTP1B overexpression could significantly elevate the level of p-AKT and p-ERK1/2 in GC cells. In vivo experiments further showed that expression of p-AKT and p-ERK1/2 was upregulated by PTP1B overexpression. These results indicated that AKT and ERK may be implicated in the miR-338-3p–PTP1B axis in GC.

For the reason why miR-338-3p was downregulated in human cancer, former studies presented two possible explanations. One was the histone methylation in the promoter region in NSCLC^48^. The other was DNA methylation in GC^51^. This difference indicated us that there may be different regulation mechanisms of miR-338-3p in different tissues. EMT refers to the alteration in morphology from epithelial like to mesenchymal like. EMT is a key process that is usually activated during cancer progression and dissemination^52^. Recent studies demonstrated that miR-338-3p exerted functions by modulating EMT progress in some kinds of tumors^48, 53^. In this article, we also found that miR-338-3p had an important impact on the migration of GC cells and peritoneal metastasis in vivo. Thus, we hypothesized that miR-338-3p could inhibit the EMT progress through suppressing PTP1B, which further damaged the capability of metastasis of GC.

In summary, this study demonstrated the critical role of PTP1B in GC. We further revealed the correlation and interaction between PTP1B and miR-338-3p in GC. The miR-338-3p–PTP1B axis generated a significant effect on cells migration and apoptosis in vitro and tumor growth and dissemination in vivo. More research needs to be conducted on PTP1B and miR-338-3p for future GC molecular therapeutics.

## Materials and methods

### Cell culture and human tissue specimens

Human GC cell lines MKN45 and MGC803 were purchased from the Institute of Biochemistry and Cell Biology, Chinese Academy of Sciences (Shanghai, China). Human gastric epithelial cell line GES-1 was purchased from Xiangya School of Medicine. All of these three cell lines were maintained in RPMI-1640 media (Gibco, USA) supplemented with 10% fetal bovine serum (FBS) at 37 °C with 5% CO_2_.

Human GC and matched adjacent normal tissues were collected from 12 pathologically confirmed GC patients who underwent curative (R0) gastrectomy in Nanjing Drum Tower Hospital. Samples were frozen in liquid nitrogen and stored at − 80 °C soon after surgery. The patient information was listed in Additional file 7: Table S1. This study was approved by the Ethics Committee of Nanjing Drum Tower Hospital.

### Western blotting

Total protein from cells and tissues was isolated using RIPA lysis buffer (Beyotime, Shanghai, China) with phenylmethylsulfonyl fluoride (Beyotime). Forty micrograms of protein extractions were resolved by 10% SDS–PAGE and transferred onto polyvinylidene difluoride membranes (Roche Diagnostics, IN, USA). The membranes were blocked with 5% non-fat dry milk and then incubated with primary antibody: anti-PTP1B and anti-GAPDH (sc-14021 and sc-47724, respectively; Santa Cruz, Dallas, TX, USA); anti-β-actin (ab8227; Abcam, Cambridge, UK); anti-Caspase-3, anti-cleaved Caspase-3, anti-AKT, anti-p-AKT, anti-ERK1/2, anti-p-ERK1/2 (9662, 9664, 9272, 4060, 4695, 4370, respectively; Cell Signaling Technology, Inc., MA, USA). Bound antibodies with anti-rabbit secondary antibodies (Santa Cruz) were detected and visualized by Pierce chemiluminescent substrate (Thermo Fisher). Photographs were taken by FLICapture (Tanon, Shanghai, China) and analyzed by ImageJ 1.50i software.

### Quantitative reverse-transcription PCR

Cells and tissues were lysed using Trizol Reagent (Invitrogen, CA, USA) following the standard procedures. To detect mature miR-338-3p, specific TaqMan miRNA Assay Probes (Applied Biosystems, Foster City, CA) were constructed. U6 snRNA served as the endogenous control. We calculated the comparative 2^-∆∆Ct^ for relative quantification in which ΔΔ*C*_T_ = (*C*_T miR-338-3p_ − *C*_T U6_)_tumor_ − (*C*_T miR-338-3p_ − *C*_T U6_)_control_.

For quantification of PTP1B and GAPDH mRNA, the RNA was reversely transcribed into cDNA with oligo d(T)18 primers (TaKaRa). Then, the quantitative reverse-transcription PCR was conducted using SYBR Green dye (Invitrogen). The specific primer sequences of PTP1B and GAPDH were as follows: PTP1B (forward): 5′-TTCTGAGCTGGGCTTGTTGT-3′; PTP1B (reverse): 5′-TGCAGCTAAAATGCAAACCCAT-3′; β-actin (forward): 5′-GCACCACACCTTCTACAATG-3′; β-actin (reverse): 5′-TGCTTGCTGATCCACATCTG-3′; GAPDH (forward): 5′- CGAGCCACATCGCTCAGACA-3′; and GAPDH (reverse): 5′- GTGGTGAAGACGCCAGTGGA-3′. β-Actin or GAPDH served as the endogenous control. The PTP1B mRNA were relatively quantified with the 2^−^^ΔΔCt^ method similar to that described above.

### Plasmids and RNA interference

Mammalian expression plasmids and an empty plasmid (control plasmid) were purchased from Genescript (Nanjing, China). siRNA against PTP1B and scrambled siRNA (control siRNA) were purchased from RioboBio (Guangzhou, Guangdong, China). The sequences of siRNA against PTP1B were as follows: 5′-GGGTCTTGCTGTAACTCAG-3′. The overexpression plasmids and siRNAs were transfected into GC cells in six-well plates using Lipofectamine 3000 (Invitrogen). Total RNA and protein were extracted at 48 h post transfection.

### miRNA overexpression and knockdown

miRNA mimics or inhibitors were respectively used to overexpress or knock down miR-338-3p expression in GC cells. miR-338-3p mimics, inhibitors, and scrambled negative control RNAs were purchased from RioboBio. MKN45 and MGC803 cells were seeded into six-well plates. At 70% confluence, cells were transfected with 200 pmol of miRNA mimic, inhibitor, or corresponding negative control using Lipofectamine 2000 (Invitrogen). At 24 h, cells were collected for total RNA or protein extraction. miR-338-3p overexpression lentivirus was also used to simulate high expression of endogenous miR-338-3p. At 40% confluence, MKN45 and MGC803 were infected with miR-338-3p lentivirus or negative control lentivirus at 20 multiplicity of infection. After 48 h, cells were collected for total RNA and protein extraction.

### Luciferase reporter assay

The wild-type segment of 3′-UTR of PTP1B containing two predicted miR-338-3p target sites (5′-ATGCTGG-3′ and 5′-TGCTGGA-3′) corresponding to the seed sequence of miR-338-3p. The mutant segment replaced the target sites by 5′-TACGACC-3′ and 5′-ACGACCT-3′, respectively. The wild-type or mutant segments were cloned into the luciferase reporter plasmid (Genescript) to conduct pMIR-PTP1B-wt-3′-UTR or pMIR-PTP1B-mut-3′-UTR. MKN45 cells in 24-well plates were co-transfected with 400 ng of either pMIR-PTP1B-wt-3′-UTR or pMIR-PTP1B-mut-3′-UTR, and 400 ng of β-galactosidase (β-gal) plasmid (Ambion), along with 50 pmol of miR-338-3p mimic, miR-338-3p inhibitor, or the corresponding negative control RNAs. The β-gal plasmid served as an internal control for normalizing transfection efficiency and cell viability. Luciferase assay was performed after 24 h, using the Luciferase Assay System (Promega, Madison, WI, USA).

### Cell migration assay

Transwell migration assays and scratch-wound assays were performed to evaluate migration of GC cells. For the transwell migration assays, MKN45 or MGC803 cells (5 × 10^4^) resuspended in 200 μl serum-free RPMI-1640 was added to the upper chamber of 24-well Transwell inserts (Millipore) containing an 8 μm pore membrane. The lower chamber was added with 500 μl RPMI-1640 plus 20% FBS. After 24 h, the Transwell inserts were fixed with 4% paraformaldehyde for 15 min. Then, the membrane was stained with 0.5% crystal violet for 15 min. A cotton swab was used to scrape the remaining cells in the upper chamber. The lower surfaces were captured using the microscope (BX51 Olympus, Japan). Five fields in each chamber were randomly chosen for quantification of migrated cells.

For the scratch-wound assays, cells were cultured in six-well plates until confluent. A scratch was created in a monolayer of transfected cells by a 200 μl pipet tip. We then removed the cellular debris and added media with 2% FBS into the plate. Serial images were taken at 0, 12, and 24 h from at least five independent fields to evaluate the wound closure. Migration was calculated as a ratio of the area covered by the cells to the original wound area.

### Cell apoptosis assay

Annexin V-FITC Apoptosis Detection Kit (BD Biosciences) was used in this assay. MKN45 or MGC cells were transfected with miR-338-3p mimic, miR-338-3p inhibitor, PTP1B-overexpression plasmid, or PTP1B siRNA for 24 h. Then, the cells were treated with serum-free RPMI-1640 medium for 24 h to induce apoptosis. Next, the cells were stained with FITC–Annexin V and propidium iodide for 15 min in the dark. The samples were finally tested using a flow cytometer (BD Biosciences) and the apoptosis populations were calculated by FlowJo X software.

### Mouse-xenograft experiments and in vivo imaging

To generate stable luciferase-positive MKN45 cell line (MKN45-Luc), MKN45 cells were infected with lentiviruses expressing firefly luciferase (GeneChem, Shanghai, China) and selected for puromycin resistance.

Six-week-old male BALB/c nude mice (Model Animal Research Center of Nanjing University, Nanjing, China) were used to establish the orthotopic transplantation model of human GC. MKN45-Luc cells were infected with miR-338-3p lentivirus to established miR-338-3p-overexpressing stable cell line. Then, MKN45-Luc cells infected with control lentivirus, miR-338-3p lentivirus, transfected with PTP1B plasmid, or co-transfected with miR-338-3p lentivirus and PTP1B plasmid, were collected (1 × 10^6^) and resuspended in phosphate-buffered saline (PBS) (10 μl) mixed with Matrigel Matrix High Concentration in 1:1 ratio (Corning, NY, USA). After intraperitoneal anesthesia with 7% chloral hydrate, a midline vertical incision was made in the upper abdomen and stomach was exposed to the outside of the peritoneal cavity. Ten-microliter cells (1 × 10^6^) in a Matrigel Matrix suspension were injected slowly using a 1 ml syringes into the subserosal area of the stomach. Then, the stomach was carefully repositioned back to the peritoneal cavity and the abdominal wall and abdominal skin were subsequently closed with interrupted vicryl sutures. The cancer growth was monitored for 3 weeks.

To establish the peritoneal dissemination model of GC, MKN45-Luc cells (6 × 10^6^ cells) suspended in 1 ml PBS were injected into the left lower peritoneal cavity (i.p.) of 6-week-old male BALB/c nude mice. The cancer growth was monitored for 3 weeks.

For fluorescence detection, mice were injected intraperitoneally with D-luciferin potassium salt (PerkinElmer, Waltham, MA, USA) dissolved in sterile PBS at a dose of 150 mg/kg body weight. Ten minutes later, bioluminescence imaging was detected using IVIS Lumina XR (PerkinElmer). Fluorescence intensity was analyzed to quantify luciferase activity of tumor tissue, which represented the size of tumors. After mice were killed, tumor samples were collected for RNA and protein extraction, H&E staining, and immunohistochemical staining for PTP1B and PCNA. All animal procedures were approved by the Bioethics Committee of Nanjing Drum Tower Hospital in compliance with international guidelines.

### Histology and immunohistochemical staining

The tumors were fixed in 4% paraformaldehyde, embedded in paraffin, and cut into 4 μm sections. H&E staining was performed following the standard procedures. Immunohistochemical staining was performed according to the manufacturer’s protocol using the anti-PTP1B, anti-PCNA antibody (1:50 dilution, Santa Cruz), and anti-cleaved Caspase-3, anti-p-AKT, and anti-p-ERK1/2 (1:50 dilution, Cell Signaling Technology, Inc.)

### Statistical analysis

All experiments were repeated at least three times. Statistical analyses were performed using SPSS 19.0 (Chicago, IL, USA) or GraphPad Prism version 7.0. The data of all experiments were presented as mean ± SE of at least three independent experiments. Statistical analysis between groups was analyzed using the Student’s *t*-test (two-tailed). Statistical differences were termed as *P* < 0.05.

## Electronic supplementary material

Additional file 1: Figure S1

Additional file 2: Figure S2

Additional file 3: Figure S3

Additional file 4: Figure S4

Additional file 5: Figure S5

Additional file 6: Figure S6

Additional file 7: Table S1

Supplementary figure legends
