# Supplementary figures and images for: miR-338-3p functions as a tumor suppressor in gastric cancer by targeting PTP1B
Source: Cell Death Dis. 2018 May 9;9(5):522. doi: 10.1038/s41419-018-0611-0 (PMC5943282; doi:10.1038/s41419-018-0611-0)

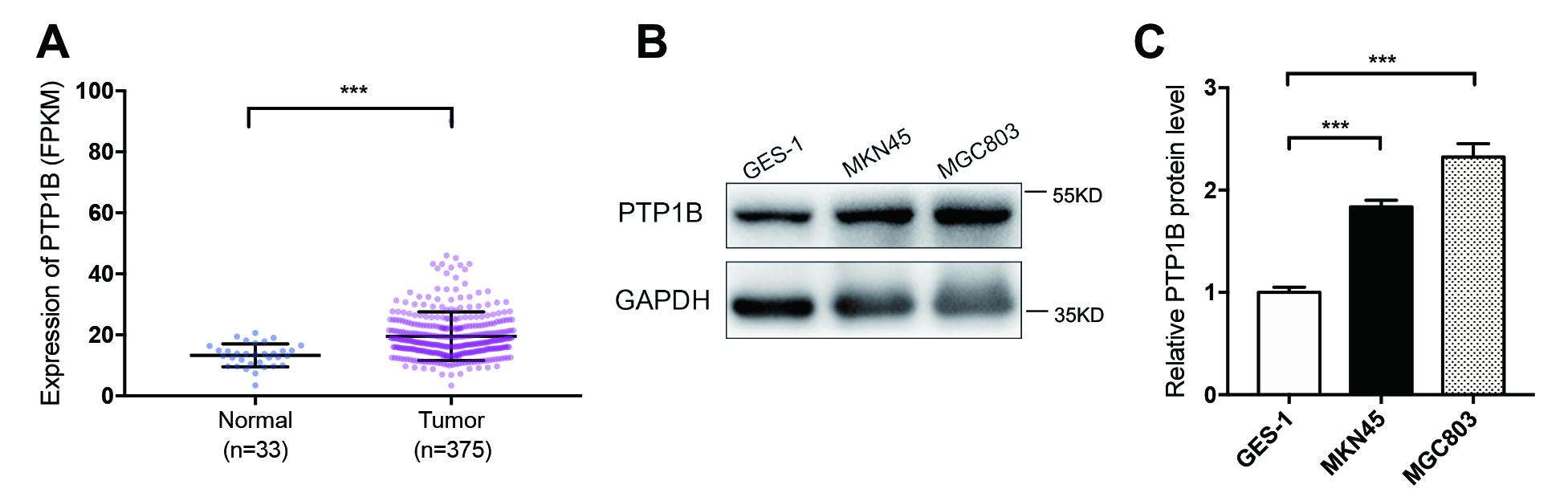

Supplement: Supplementary file 1 — Additional file 1: Figure S1 [file 41419_2018_611_MOESM1_ESM.tif]

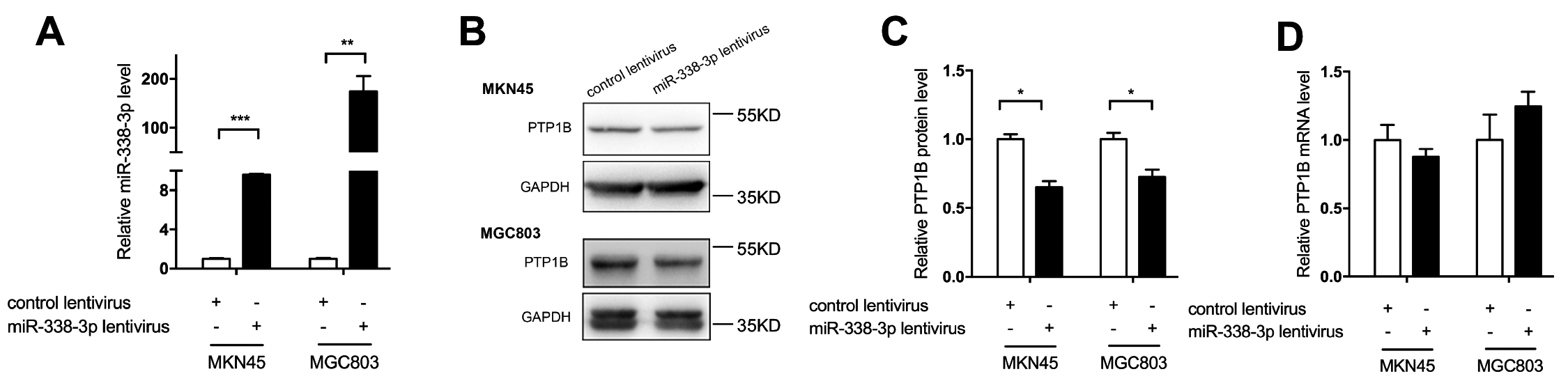

Supplement: Supplementary file 2 — Additional file 2: Figure S2 [file 41419_2018_611_MOESM2_ESM.tif]

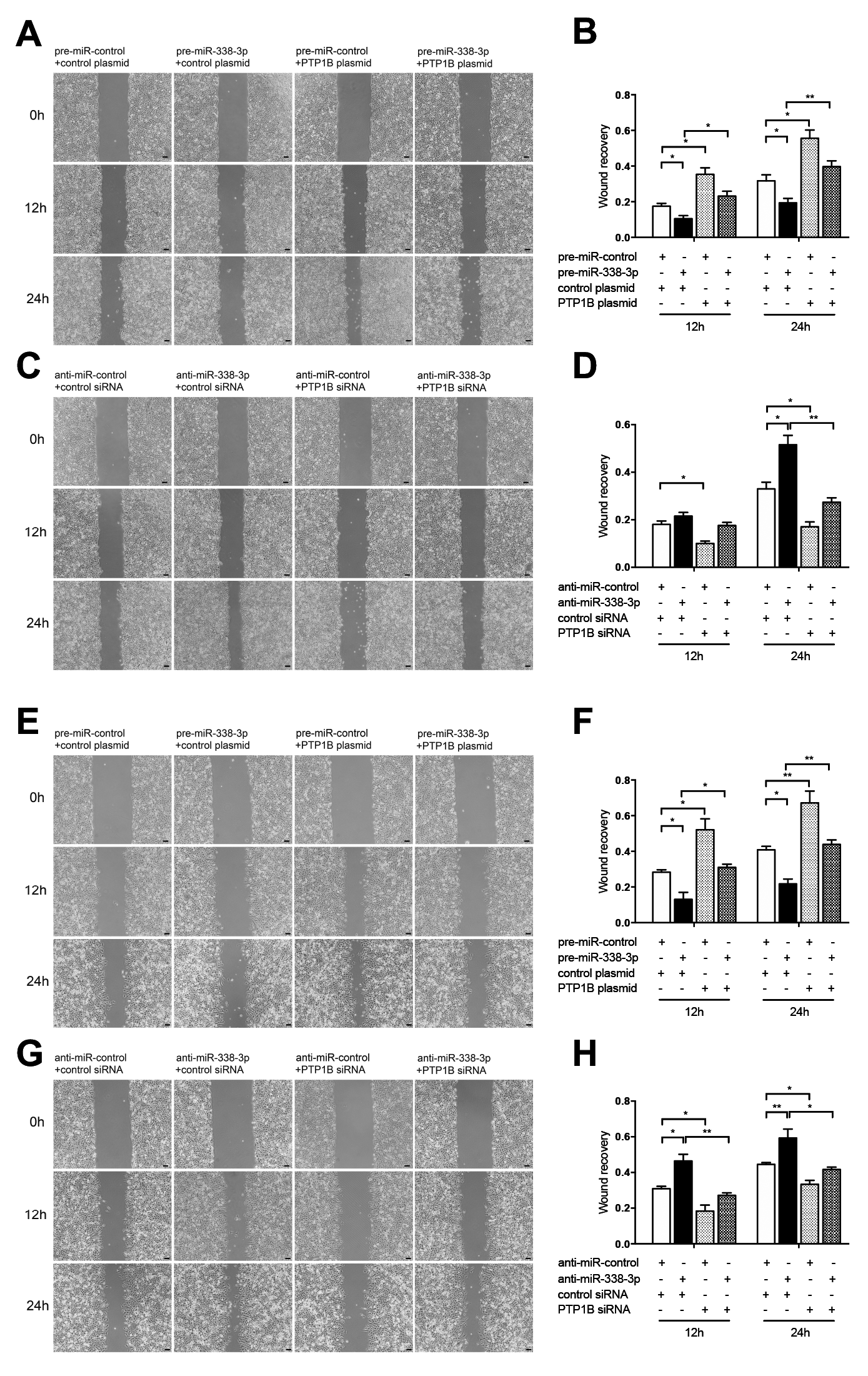

Supplement: Supplementary file 3 — Additional file 3: Figure S3 [file 41419_2018_611_MOESM3_ESM.tif]

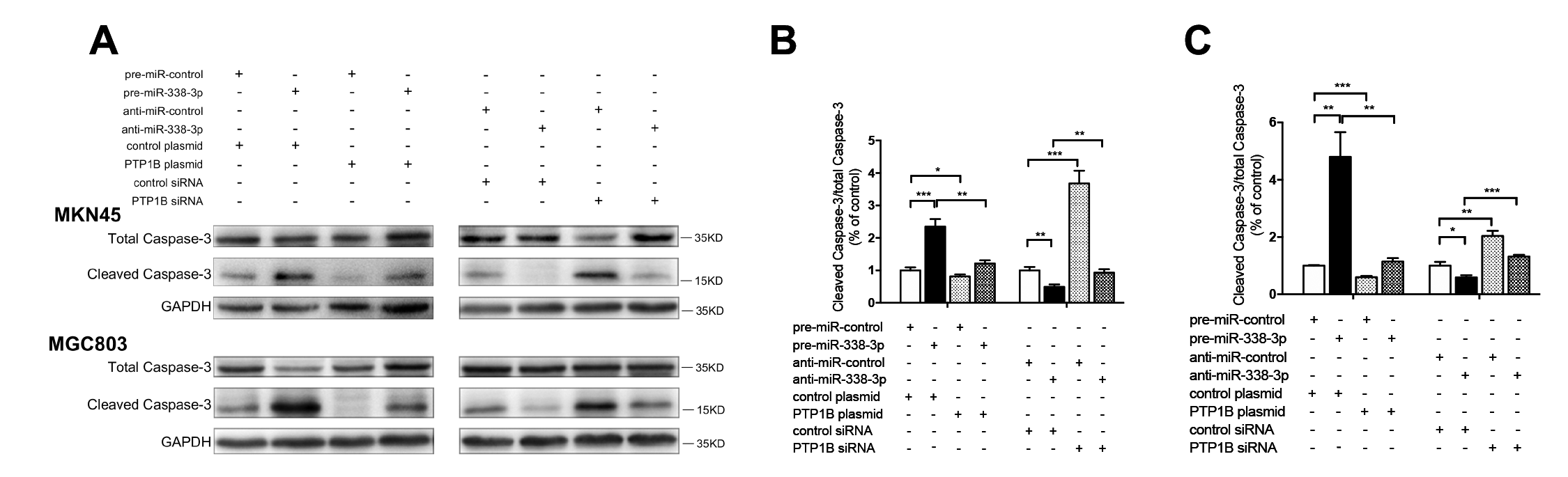

Supplement: Supplementary file 4 — Additional file 4: Figure S4 [file 41419_2018_611_MOESM4_ESM.tif]

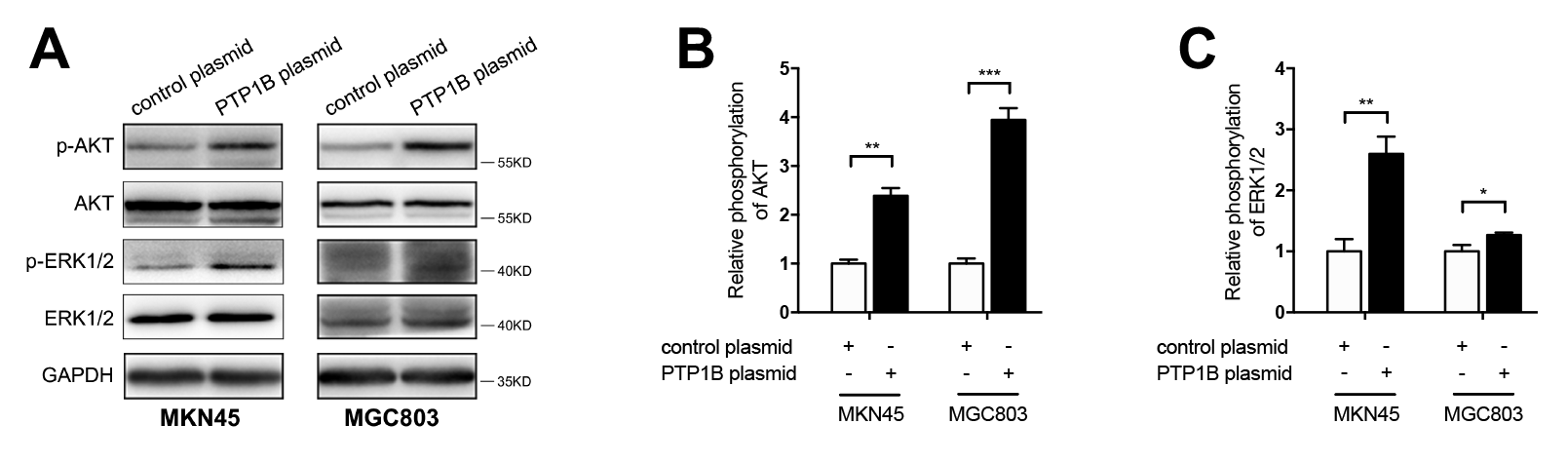

Supplement: Supplementary file 5 — Additional file 5: Figure S5 [file 41419_2018_611_MOESM5_ESM.tif]

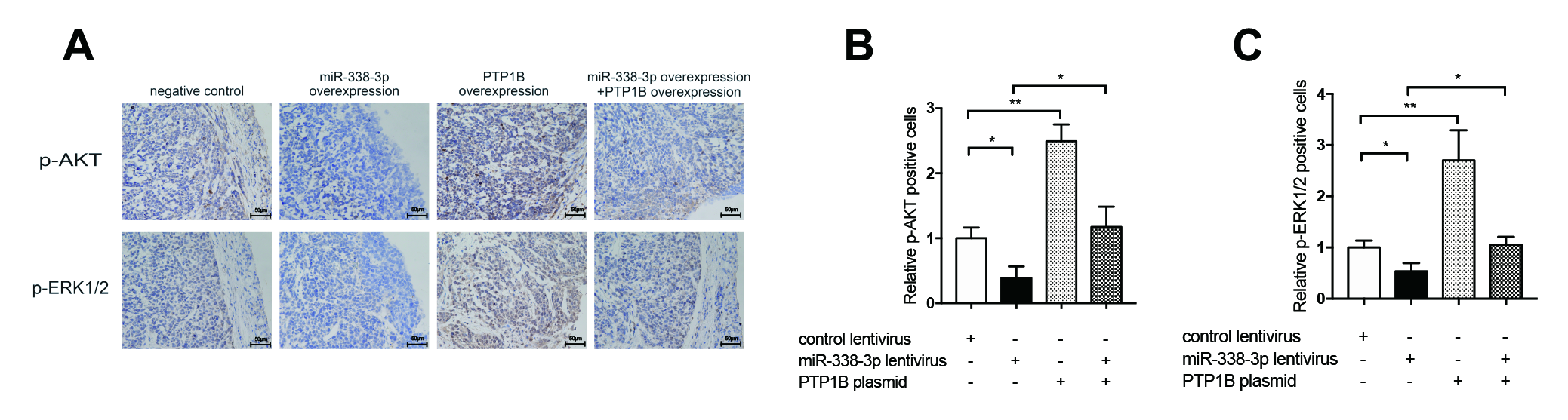

Supplement: Supplementary file 6 — Additional file 6: Figure S6 [file 41419_2018_611_MOESM6_ESM.tif]
