# Supplementary material for: miR-338-3p functions as a tumor suppressor in gastric cancer by targeting PTP1B
Source: Cell Death Dis. 2018 May 9;9(5):522. doi: 10.1038/s41419-018-0611-0 (PMC5943282; doi:10.1038/s41419-018-0611-0)
Supplement: Supplementary file 7 — Additional file 7: Table S1 [file 41419_2018_611_MOESM7_ESM.docx]

**Additional file 1: Table S1.** **Clinical features of gastric cancer patients.**

| Case No. | Age（years） | Gender | TNM Stage | Clinical History |
| --- | --- | --- | --- | --- |
| 1 | 45 | female | ⅢB | Gastric Cancer |
| 2 | 61 | male | ⅡA | Gastric Cancer |
| 3 | 73 | male | ⅡB | Gastric Cancer |
| 4 | 52 | female | ⅢA | Gastric Cancer |
| 5 | 61 | female | ⅢB | Gastric Cancer |
| 6 | 74 | male | ⅡB | Gastric Cancer |
| 7 | 65 | male | ⅢC | Gastric Cancer |
| 8 | 68 | female | Ⅳ | Gastric Cancer |
| 9 | 55 | male | ⅢC | Gastric Cancer |
| 10 | 53 | male | ⅡB | Gastric Cancer |
| 11 | 67 | female | ⅢB | Gastric Cancer |
| 12 | 78 | male | ⅡA | Gastric Cancer |
