# Supplementary material for: miR-338-3p functions as a tumor suppressor in gastric cancer by targeting PTP1B
Source: Cell Death Dis. 2018 May 9;9(5):522. doi: 10.1038/s41419-018-0611-0 (PMC5943282; doi:10.1038/s41419-018-0611-0)
Supplement: Supplementary file 8 — Supplementary figure legends [file 41419_2018_611_MOESM8_ESM.docx]

**Additional file 1:** **Figure S1.** PTP1B is up-regulated in GC. **(a)** Expression of PTP1B in GC samples from TCGA. (n=408). Data are represented as mean ± SD. **(b,c)** Western blot analysis of PTP1B protein in GES-1, MKN45, and MGC803 cells. **(b)** representative images; **(c)** quantitative analysis. Data are represented as mean ± SEM. *** p < 0.001; two-tailed Student’s t-test.

**Additional file 2:** **Figure S2.** PTP1B protein level is suppressed in GC cells infected with miR-338-3p lentivirus. (**a-d)** MKN45 and MGC803 cells were infected with miR-338-3p lentivirus and control lentivirus. After 48 h, cells were harvested for total RNA and protein extraction. (**a)** qRT-PCR analysis of miR-338-3p levels in MKN45 and MGC803 cells. (**b**,**c)** Western blot analysis of PTP1B protein in MKN45 and MGC803 cells. (**b)** representative images; (**c)** quantitative analysis. (**d)** qRT-PCR analysis of PTP1B mRNA levels in MKN45 and MGC803 cells. n=3 independent repeats; Data are represented as mean ± SEM; *P < 0.05; **P < 0.01; ***P < 0.001; two-tailed Student’s *t*-test.

**Additional file 3: Figure S3.** miR-338-3p suppresses GC cells migration by targeting PTP1B. The scratch-wound assays were performed in MKN45 (**a-d)** and MGC803 (**e-h)** cells that were transfected with control mimic plus control plasmid, miR-338-3p mimic plus control plasmid, control mimic plus PTP1B overexpression plasmid, miR-338-3p mimic plus PTP1B overexpression plasmid, or with control inhibitor plus control siRNA, miR-338-3p inhibitor plus control siRNA, control inhibitor plus PTP1B siRNA, miR-338-3p inhibitor plus PTP1B siRNA. Images were taken at 0, 12 and 24 h after the scratch was applied to evaluate the wound closure. (**a,c)** representative images of MKN45 cells; Scale bar, 100 μm. (**b,d)** quantitative analysis. (**e,g)** representative images of MGC803 cells; Scale bar, 100 μm. (**f,h)** quantitative analysis. n=3 independent repeats; Data are represented as mean ± SEM; *P < 0.05; **P < 0.01; two-tailed Student’s *t*-test.

**Additional file 4: Figure S4.** miR-338-3p promotes GC cell apoptosis by targeting PTP1B. (**a-c)** Western blot analysis of cleaved Caspase-3 protein in MKN45 and MGC803 cells transfected with control mimic plus control plasmid, miR-338-3p mimic plus control plasmid, control mimic plus PTP1B overexpression plasmid, miR-338-3p mimic plus PTP1B overexpression plasmid, or with control inhibitor plus control siRNA, miR-338-3p inhibitor plus control siRNA, control inhibitor plus PTP1B siRNA, miR-338-3p inhibitor plus PTP1B siRNA. (**a)** representative images; (**b)** quantitative analysis of MKN45 cells; (**c)** quantitative analysis of MGC803 cells. n=3 independent repeats; Data are represented as mean ± SEM; *P < 0.05; **P < 0.01; ***P < 0.001; two-tailed Student’s *t*-test.

**Additional file 5: Figure S5.** Effect of PTP1B on the expression of AKT and ERK1/2. (**a-c)** Western blot analysis of total and phosphorylated AKT and ERK1/2 protein in MKN45 and MGC803 cells transfected with control or PTP1B plasmid. (**a)** representative images; (**b)** quantitative analysis of phosphorylation of AKT; (**c)** quantitative analysis of phosphorylation of ERK1/2. n=3 independent repeats; Data are represented as mean ± SEM; * p < 0.05; ** p < 0.01; *** p < 0.001; two-tailed Student’s *t*-test.

**Additional file 6: Figure S6.** Immunohistochemical staining for p-AKT and p-ERK1/2 in orthotopic transplantation tumors. (**a)** representative images; Scale bar, 50 μm. (**b,c)** quantitative analysis. n=10 mice per group; Data are represented as mean ± SEM; * p < 0.05; ** p < 0.01; two-tailed Student’s *t*-test.

**Additional file 7:** **Table S1.** Patients’ Characteristics.
